# MAGEA Family Gene Expression Influences Survival in Intestinal‐Type Gastric Cancer

**DOI:** 10.1002/cam4.72298

**Published:** 2026-09-17

**Authors:** Silke Neumann, Trevor Leong, Rita A. Busuttil, Alex Boussioutas, Sharon T. Pattison

**Affiliations:** ^1^ Department of Pathology and Molecular Medicine, Faculty of Medicine–Dunedin University of Otago Dunedin New Zealand; ^2^ Department of Radiation Oncology Peter MacCallum Cancer Centre Melbourne Victoria Australia; ^3^ Sir Peter MacCallum Department of Oncology University of Melbourne Melbourne Victoria Australia; ^4^ Department of Gastroenterology School of Translational Medicine, Monash University Melbourne Victoria Australia; ^5^ Department of Gastroenterology The Alfred Hospital Melbourne Victoria Australia; ^6^ Health New Zealand ‐ Te Whatu Ora Capital, Coast and Hutt Valley Newtown, Wellington New Zealand; ^7^ Department of Cancer Sciences Faculty of Medical and Health Sciences, University of Auckland Grafton, Auckland New Zealand

**Keywords:** gene expression profiling, MAGE‐A12 antigen human, MAGE‐A3 antigen human, progression‐free survival, stomach neoplasms

## Abstract

Gastric cancer (GC) is a heterogeneous disease characterized by diverse molecular profiles and distinct histological subtypes, each with differing prognoses and therapeutic responses. Melanoma‐associated antigen‐A (*MAGEA*) genes encode cancer testis antigens frequently expressed in tumor tissue and in limited amounts in normal tissues, making them a promising target for cancer immunotherapy. While therapies targeting MAGEA family members have demonstrated promise across various cancers, clinical trials have been limited by substantial toxicities, underscoring the need to precisely identify patient subgroups most likely to benefit from MAGEA‐targeted treatments. We performed gene expression analysis of *MAGEA3, 6* and *12* in an Australian‐based multicenter cohort of 100 GC patients, including 49 intestinal‐type GC cases accrued between 1999 and 2009, and compared expression profiles with those of the Cancer Genome Atlas (TCGA) cohort. Expression patterns were determined in histological and TCGA molecular subtypes of GC. Progression‐free survival was examined using the log‐rank test and Cox proportional hazards modeling. *MAGEA3, 6*, and *12* were mainly expressed in intestinal‐type GC and were not associated with other clinicopathological variables. Multivariate analyses revealed that elevated expression of these MAGEA members was prognostic of poorer survival in intestinal‐type GC patients. In the TCGA cohort, higher *MAGEA3, 6*, and *12* expression was observed in intestinal‐type GC and the chromosomal instability (CIN) subtype, and associations of poorer progression‐free survival with high expression were found. Our findings highlight the differences in GC subtype biology and suggest a potential role for *MAGEA3, 6*, and *12* as biomarkers and targets for intestinal‐type GC immunotherapy; however, the relatively small intestinal‐type GC sample size warrants validation in larger cohorts.

**Abbreviations**
AJCC: American Joint Committee on Cancer
CIN: chromosomal instability
EBV: Epstein–Barr virus
GC: gastric cancer
GEO: Gene Expression Omnibus
GS: gfenomically stable
MAGEA: melanoma‐associated antigen‐A
MSI: microsatellite instability
PFS: progression‐free survival
TCGA: Cancer Genome Atlas

## 1 Introduction

Gastric cancer (GC) is the fifth most common cause of cancer and is associated with a high mortality rate [1]. Risk factors associated with the development of GC are infections ( *Helicobacter pylori* , Epstein–Barr virus), chronic inflammation and genomic instability [2, 3]. Symptoms of GC can easily be mistaken as indigestion or discomfort, leading to a late diagnosis of the cancer. Epidemiological and biological evidence suggest that GC is heterogeneous and that treatment responses differ in subtypes of GC [4, 5, 6, 7, 8]. However, despite the heterogeneity, GC is treated as one disease.

GC can be classified based on histological appearance, anatomical location and molecular signatures [2, 9, 10, 11, 12, 13, 14, 15]. The most commonly used histological classification systems include the Laurén and World Health Organization classifications. Based on microscopic appearance, two main histological subtypes were defined by Laurén in 1965 [16], intestinal and diffuse‐type GC. Intestinal‐type GCs show a well‐developed tissue architecture with closely connected cancer cells that form glands. The tissue structure is supported by cancer cells adhering to neighboring cells and anchoring of these cancer cell agglomerates in the stomach wall. In contrast, diffuse‐type gastric cancer presents as solitary and poorly cohesive cancer cells infiltrating diffusely in the gastric wall without gland formation, cell adhesion and mechanical links [16]. Tumors that have features of both subtypes were described as mixed.

Treatment of advanced GC is challenging and entails chemo‐ and radiation therapy and surgery, which achieve modest survival benefits. In the recent past, evidence of efficacy for immune checkpoint inhibitors has been demonstrated in some advanced GCs, but the benefit is modest [17, 18, 19, 20]. Therefore, there is a pressing need to develop novel therapies to benefit GC patients. Immunotherapy is a promising approach to treat GC by targeting GC cells expressing unique tumor‐associated antigens absent or expressed at lower levels in normal cells. An interesting target is cancer testis antigens, expressed in the testis, placenta and various tumor types. The MAGEA family of genes is a sub‐family of cancer testis antigens initially identified in melanoma, and subsequently found to be expressed in germ cells, a limited number of normal tissues, and on several different cancers, including GC [21, 22]. *MAGEA* genes are expressed in GC tissues and have been associated with poorer patient survival and clinicopathological features in several patient cohorts [21, 23, 24, 25, 26].

The MAGEA family includes 11 genes encoded on the X chromosome [27]. The coding sequence is highly conserved within the family, with only 12 of 314 (3.8%) amino acids varying in sequence between *MAGEA3* and *MAGEA6*; this close homology has limited the ability to generate specific antibodies to particular family members [27]. In normal tissues, MAGEA family expression is repressed by methylation of promoter regions; in cancers, hypomethylation of these promoters is one of the mechanisms by which these genes become expressed, and this has been demonstrated in GC [28].

The differential expression of MAGEA family genes and proteins in normal and cancer tissues makes them attractive targets for cancer immunotherapy. MAGEA family members stimulated anti‐tumor immune responses in clinical trials; however, due to unrecognized MAGEA expression in the brain, severe toxicities, including neurotoxicity leading to death, occurred [27, 29, 30, 31, 32]. Several studies demonstrated the feasibility of targeting MAGEA family members and achieving isolated partial or complete responses in metastatic cancer [33, 34, 35]. An overview of trials is provided in this review [36]. Recently, the Food and Drug Administration approved a diagnostic test to detect MAGEA4 expression in patients with synovial sarcoma and a T‐cell therapeutic targeting MAGEA4 has shown promise for the treatment of synovial sarcoma and liposarcoma in a Phase II clinical trial [37]. These studies highlight the promise of MAGEA family members as therapeutic targets and the need to consider their application for different types of cancer carefully.

Since GC is heterogeneous, we assessed *MAGEA* family expression and their influence on patient survival in histological and molecular subtypes of GC to determine whether expression levels differ and if MAGEA could be a potential target for cancer therapy in subtypes of GC. Expression of *MAGEA3, 6*, and *12* was assessed in tumor and normal tissue in an Australian cohort (Molecular Analysis of Upper Gastro‐intestinal Cancer, MAUGIC [4]) and in publicly available cohorts. MAUGIC is an ongoing tissue banking study from selected Melbourne hospitals from 1999 to the present. Our findings provide insights into the expression of *MAGEA3, 6*, and *12* in subtypes of GC, their link with clinicopathological features, and how they could be utilized as potential therapeutic targets.

## 2 Materials and Methods

### 2.1 Gastric Cancer Cohort and Tissue Collection

Gastric cancer and normal tissue were collected at selected Melbourne hospitals from patients enrolled in the MAUGIC cohort as described previously [4]. Written informed consent was obtained from study participants, and ethical approval was obtained from Institutional Review Boards from individual hospitals associated with the study (Peter MacCallum Cancer Centre 12/25). Study investigators identified patients before surgery and collected demographic and clinical information. As an observational cohort, no influence was made on patient care.

Tumor and normal tissue were collected at the time of surgery. Normal tissue was collected from a region at least 2 cm from the primary tumor in the same anatomic region of the stomach. A pathological review of the submitted tumor and normal tissue was performed to confirm the presence of tumor, histological subtype of tumor, and any associated intestinal metaplasia or chronic gastritis. Resected tumors were reviewed by local pathologists, and the stage was reported using the American Joint Committee on Cancer (AJCC) 6th edition TNM staging system [38]. Tumor location was identified macroscopically at pathological assessment, from operative notes or pre‐operative investigations. Tumors included in this analysis were limited to adenocarcinomas. Patients enrolled up to 2009 were included in this study, with the last survival data update being in 2012.

### 2.2 Transcriptomic Analysis

Tumor and normal tissue samples were fresh frozen at the time of surgery and gene expression data was generated using Affymetrix Human Genome U133 plus 2.0 GeneChips (HG‐U133 + 2.0) for 100 tumor and 52 matched normal specimens. All tumor samples had at least 60% tumor cells and no tumors were excluded due to low percentage of tumor cells. Total ribonucleic acid (RNA) was isolated by acid phenol extraction (Trizol, Invitrogen) and RNeasy mini kit (Qiagen). The data has previously been submitted to the Gene Expression Omnibus (GEO, Series GSE51105) [39, 40]. Probe sets for *MAGEA3, 6*, *and 12* were examined using GeneAnnot for the sensitivity and specificity of individual probe sets [41]. Other MAGE family members were not evaluated because corresponding probe sets were not available on the Affymetrix array. When more than one probe set was present, the probe set with the highest sensitivity and specificity was chosen. *MAGEA3, 6*, and *MAGEA12* expression was dichotomised at 8 and 7.5, respectively, based on visual assessment of the density inflection point. The Cancer Genome Atlas (TCGA) data were accessed via the cBioPortal interface [42, 43]. The Kaplan–Meier (km) plotter was used to interrogate gastric cancer Affymetrix datasets on https://kmplot.com/analysis/.

Differential gene expression was examined using R with Bioconductor (www.bioconductor.org) packages affy and limma [44, 45, 46, 47]. Raw CEL files were imported into R and normalized using robust multi‐array average to generate the expression set for analyses [48]. Differential gene expression analyses were performed using a modified *t*‐test in limma, adjusted using the Benjamini Hochberg correction [49].

### 2.3 Statistical Analysis

Progression‐free survival (PFS) was measured from the date of surgery to the date of first documented relapse, progression, or death from gastric cancer if recurrence was not documented. Progression‐free survival was chosen instead of recurrence‐free survival, as the subset of patients for whom gene expression data is available includes six patients who underwent palliative resections and two patients for whom metastases were identified at surgery. Progression was defined as radiological, histopathological, or clinical evidence of recurrence or progression of measurable disease. The survival analysis was performed using the Survival package in R, and comparisons were performed using a two‐sided log‐rank test [50, 51]. Survival curves were generated using the Kaplan–Meier method. Multivariate analysis was performed using the Cox proportional hazards method, including overall AJCC stage in the model, with this having been identified as a significant predictor of survival in the MAUGIC cohort along with Lauren classification in prior analyses [4]. Other clinical variables were not included because they were available only for a limited proportion of patients and therefore could not be reliably incorporated into the model. Proportional hazards assumptions were assessed using Schoenfeld residuals. Chi‐squared test was used to examine the significance of categorical data, and the Wilcoxon or Kruskal‐Wallis rank sum test for rank data.

## 3 Results

### 3.1 *MAGEA* Family Gene Expression in Histological Subtypes of GC

**FIGURE 1:**
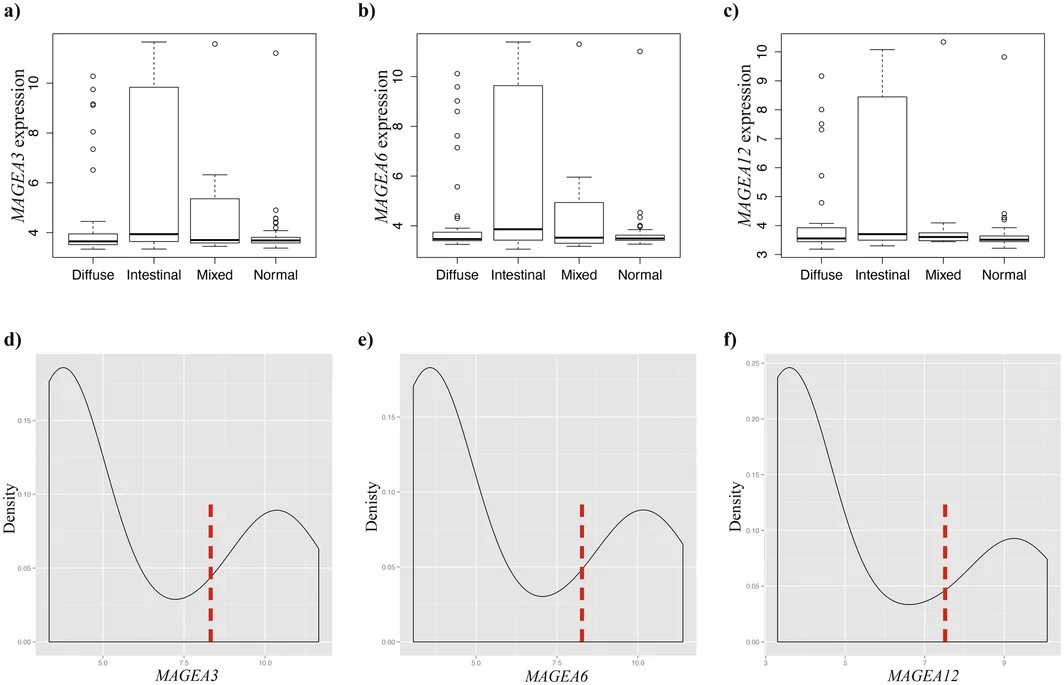
MAGEA family expression differs in histological subtypes of GC. Boxplots show (a) *MAGEA3*, (b) *MAGEA6* and (c) *MAGEA12* gene expression values in diffuse‐type (*n* = 40), intestinal‐type (*n* = 49), mixed (*n* = 11) GC and normal tissue (*n* = 52). Analysis of MAGEA family gene expression density in intestinal‐type GC is shown in (d–f), with *MAGEA3, 6* and *MAGEA12* expression dichotomised at 8 and 7.5, respectively, based on visual assessment of the density inflection point.

To understand the role of *MAGEA3, 6* and *12* expression in this Australian cohort, we analyzed their expression in GC tissue samples divided into different histological subtypes according to Laurén classification. In accordance with previous studies, *MAGEA3, 6* and *12* were found to be expressed in GC but at minimal levels in normal tissues (Figure 1). Interestingly, MAGEA genes were mainly found to be expressed in the intestinal‐type GC subtype. *MAGEA3* (adjusted *p*‐value, 0.039) and *MAGEA6* (adjusted *p*‐value, 0.048) had a 1.65‐fold and *MAGEA12* (adjusted *p*‐value, 0.01) a 1.27‐fold higher expression in intestinal‐type GC as compared with diffuse‐type GC (Figure 1). Expression density plots of these MAGEA family member genes for intestinal‐type GC tumors revealed the majority had background levels of expression only, with one‐third of intestinal‐type GCs (16/49, 33%) having higher expression (Figure 1d–f).

### 3.2 *MAGEA* Expression and Progression‐Free Survival in an Australian GC Cohort

**FIGURE 2:**
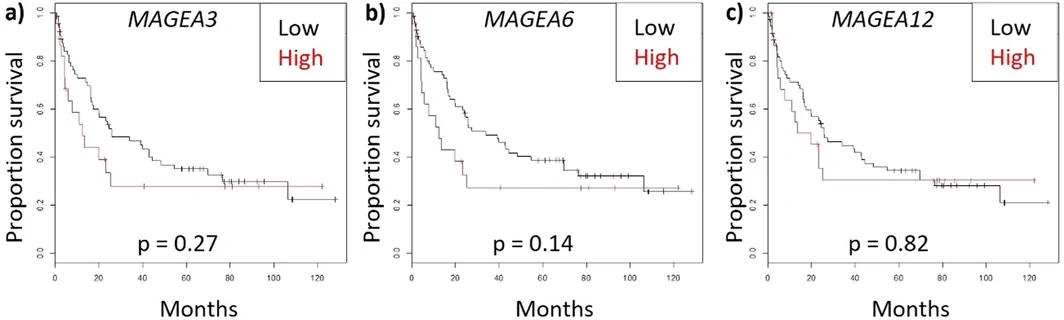
High expression of *MAGEA3, 6* and *12* is not predictive of progression‐free survival in GC patients. Gastric cancer tumor tissues (*n* = 100) were collected from the primary tumor and gene expression of (a) *MAGEA3* (high *n* = 23, low *n* = 77; *p* = 0.27), (b) *MAGEA6* (high *n* = 22, low *n* = 78; *p* = 0.14), and (c) *MAGEA12* (high *n* = 18, low *n* = 82; *p* = 0.82) was determined using the Affymetrix Human Genome U133 plus 2.0 GeneChip.

Given the bimodal distribution of expression of the MAGEA family genes, expression levels for the survival analysis were dichotomized around the inflection point of the expression density curve for each gene (8 for *MAGEA3* and *MAGEA6*; 7.5 for *MAGEA12*). In contrast to previous GC cohorts, high *MAGEA3, 6*, and *12* expression was not predictive of progression‐free survival in GC patients (Figure 2, *MAGEA3*, *p* = 0.27; *MAGEA6, p* = 0.14; *MAGEA12*, *p* = 0.82).

**FIGURE 3:**
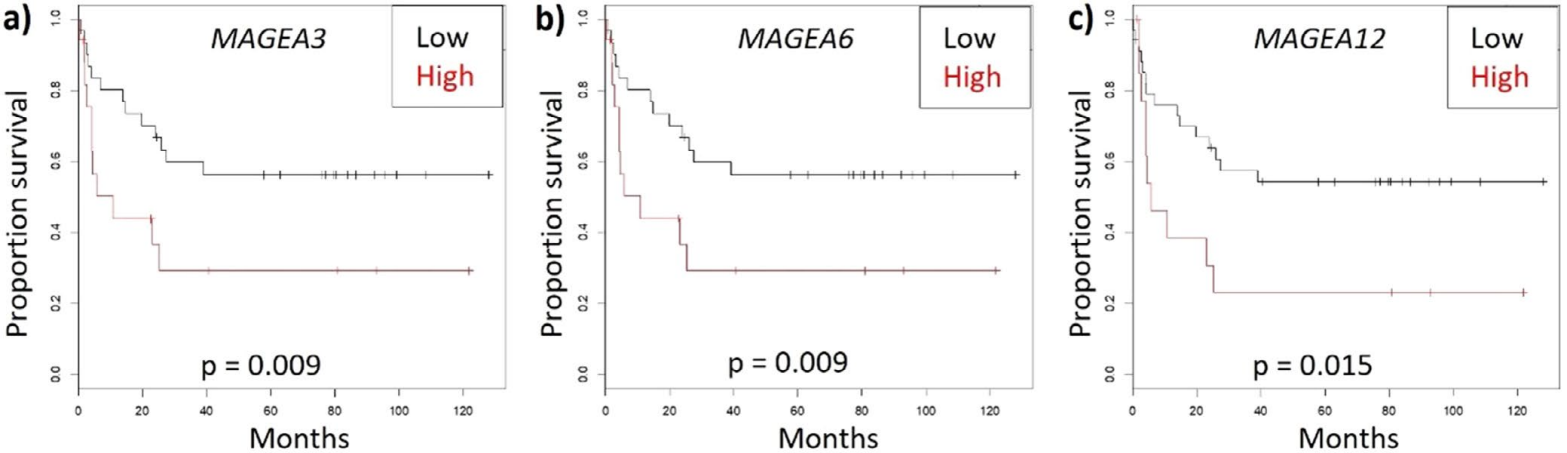
High *MAGEA* family gene expression is associated with poorer survival in intestinal‐type GC patients (*n* = 49). High (red line) and low expression (black line) of (a) *MAGEA3* (high *n* = 16, low *n* = 33; *p* = 0.009), (b) *MAGEA6* (high *n* = 16, low *n* = 33; *p* = 0.009), and (c) *MAGEA12* (high *n* = 13, low *n* = 36; *p* = 0.015) in intestinal‐type GC patients (*n* = 49) as determined by multivariate analysis using the Cox proportional hazard method.

Since *MAGEA3, 6* and *12* expression was mainly observed in intestinal‐type GC samples and not predictive of progression‐free survival in all GCs, we analyzed their effect on patient survival in the intestinal‐type of GC. All three genes were found to be predictive of progression‐free survival outcomes in intestinal‐type GC patients on univariate and multivariate analysis that included AJCC stage (Figure 3 and Table 1), multivariate analysis hazard ratio for *MAGEA3* and *MAGEA6* 3.45, 95% CI 1.35–8.79, *p* = 0.009; hazard ratio for *MAGEA12* 3.38, 95% CI 1.27–8.98, *p* = 0.015. Low *MAGEA3, 6* and *12* gene expression conferred a survival benefit to intestinal‐type GC patients, whereas patients with high *MAGEA* family gene expression had poorer survival.

**TABLE 1:** Univariate and multivariate progression‐free survival analysis based on MAGEA family gene expression, in intestinal‐type GC patients. The multivariate analysis includes AJCC stage.

Gene	*n* (%)	Univariate analysis			Multivariate analysis		
		Hazard ratio	95% CI	*p*	Hazard ratio	95% CI	*p*
*MAGEA3*							
Low	33 (67.3)	1	—		1	—	
High	16 (32.7)	2.32	1.04–5.15	0.03	3.45	1.35–8.79	0.009
*MAGEA6*							
Low	33 (67.3)	1	—		1	—	
High	16 (32.7)	2.32	1.04–5.15	0.03	3.45	1.35–8.79	0.009
*MAGEA12*							
Low	36 (73.5)	1	—		1	—	
High	13 (26.5)	2.61	1.16–5.87	0.02	3.38	1.27–8.98	0.015

### 3.3 *MAGEA* Expression in Intestinal‐Type GC Patients and Clinicopathological Features

As MAGEA gene expression has previously been associated with clinicopathological features, we examined intestinal‐type GC patients from this cohort, with high and low expression of *MAGEA3, 6*, and *12*, for differences in associated clinicopathological features. There were no significant differences in any of the clinicopathological features described in Table 2.

**TABLE 2:** Clinical and pathological characteristics by MAGEA family gene expression in intestinal‐type GC.

Variable	Low expression *n* = 33 (%)	High expression *n* = 16 (%)	*p*
Age (years)			1.00*
Median	69.0	68.5	
Range	48–85	33–74	
Gender			0.53
Female	8 (24.2)	6 (37.5)	
Male	25 (75.8)	10 (62.5)	
Tumor location			0.20
GOJ/Cardia	9 (27.3)	4 (25.0)	
Body	19 (57.6)	6 (37.5)	
Antrum	5 (15.2)	6 (37.5)	
Lymphovascular invasion			0.34
Present	18 (54.5)	12 (75.0)	
Absent	9 (27.3)	3 (18.8)	
Not documented	6 (18.2)	1 (6.2)	
Differentiation			0.64
Well	2 (6.1)	1 (6.2)	
Moderate	21 (63.6)	8 (50.0)	
Poor	10 (30.3)	7 (43.8)	
Inflammatory score			0.16
1	11 (33.3)	6 (37.5)	
2	7 (21.2)	7 (43.8)	
3	9 (27.3)	3 (18.8)	
Not documented	6 (18.2)	0 (0.0)	
T stage			0.65
T1	5 (15.2)	1 (6.2)	
T2	10 (30.3)	6 (37.5)	
T3	18 (54.5)	9 (56.2)	
N stage			0.68
N0	14 (42.4)	4 (25.0)	
N1	13 (39.4)	8 (50.0)	
N2	4 (12.1)	3 (18.8)	
N3	2 (6.1)	1 (6.2)	
AJCC stage			0.51
Stage I	8 (24.2)	4 (25.0)	
Stage II	12 (36.4)	3 (18.8)	
Stage III	10 (30.3)	8 (50.0)	
Stage IV	3 (9.1)	1 (6.2)	
Cause of death			0.42
Not applicable	13 (40.6)	5 (31.2)	
Non‐gastric cancer	5 (15.6)	1 (6.2)	
Gastric cancer	14 (43.8)	10 (62.5)	

*Note:* Chi‐squared test all *p*‐values except asterisk symbol (*) indicates Wilcoxon rank sum test.

### 3.4 MAGEA Family Gene Expression in Publicly Available Intestinal‐Type GC Datasets

To explore the association of MAGEA family gene expression with intestinal‐type GC, the expression of MAGEA family members was investigated in the published TCGA cohort [2] and additional cohorts accessible on km‐plotter (https://kmplot.com/analysis/) [52, 53].

**FIGURE 4:**
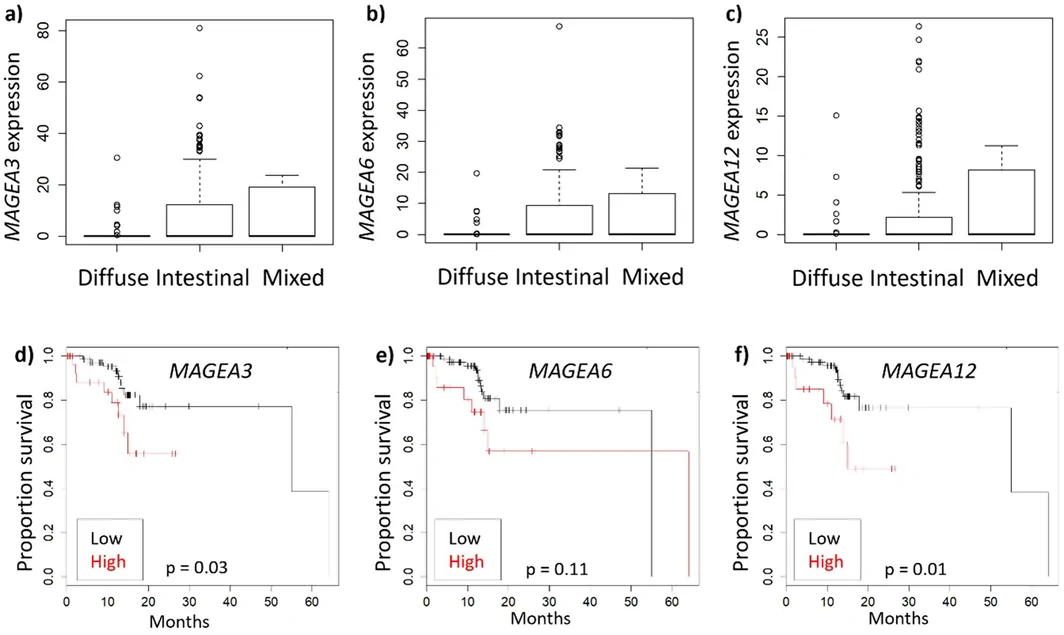
Intestinal‐type GC patients in publicly available datasets with low expression of *MAGEA* genes have improved survival. Gene expression values for (a) MAGEA3 (high *n* = 46, low *n* = 127; *p* = 0.0242), (b) MAGEA6 (high *n* = 42, low *n* = 131; *p* = 0.001), and (c) MAGEA12 (high *n* = 37, low *n* = 136; *p* = 0.068) were extracted from the TCGA gastric cancer cohort (*n* = 265) and compared using the Cox proportional hazards method. Univariate analysis of progression‐free survival revealed that low expression (black line) of (d) MAGEA3 (*p* = 0.03) and (f) MAGEA12 (*p* = 0.01) are predictive of improved progression‐free survival while (e) MAGEA6 (*p* = 0.11) expression was not.

Expression values were available for 89.8% (265/295) of all patients in the TCGA cohort, with 88.3% (173/196) of intestinal‐type GCs and 94.2% (65/69) of diffuse‐type GCs having RNA sequencing data available. Expression of *MAGEA3* (*p* = 0.0242) and *MAGEA6* (*p* = 0.001) was higher in intestinal‐type GC when compared to diffuse‐type GC in the TCGA cohort; the difference in expression was not significant in *MAGEA12* (*p* = 0.068), although expression of *MAGEA12* was lower overall (Figure 4a–c). As seen in the MAUGIC cohort, mixed GC also showed higher expression than diffuse‐type GC. The expression boxplots showed a similar distribution to the MAUGIC cohort, with most tumors having low expression and a few cases, mainly intestinal‐type GC, contributing to the higher expression subset.

Progression‐free survival was also examined, although limited follow‐up data were available at the time of this analysis. On univariate analysis of progression‐free survival, higher expression of *MAGEA3* (HR 2.75, 95% CI 1.06–7.13, *p* = 0.03) and *MAGEA12* (HR 3.22, 95% CI 1.22–8.46, *p* = 0.01) was associated with poorer survival, but *MAGEA6* (HR 2.16, 95% CI 0.83–5.65, *p* = 0.11) expression was not (Table 3 and Figure 4d–f).

**TABLE 3:** Univariate progression‐free survival analysis for MAGEA family gene expression in intestinal‐type GC, TCGA cohort.

Gene	*n* (%)	Univariate analysis		
		Hazard ratio	95% CI	*p*
*MAGEA3*				
Low	127 (73.4)	1	—	
High	46 (26.5)	2.75	1.06–7.13	0.03
*MAGEA6*				
Low	131 (75.7)	1	—	
High	42 (24.3)	2.16	0.83–5.65	0.11
*MAGEA12*				
Low	136 (78.6)	1	—	
High	37 (21.4)	3.22	1.22–8.46	0.01

**FIGURE 5:**
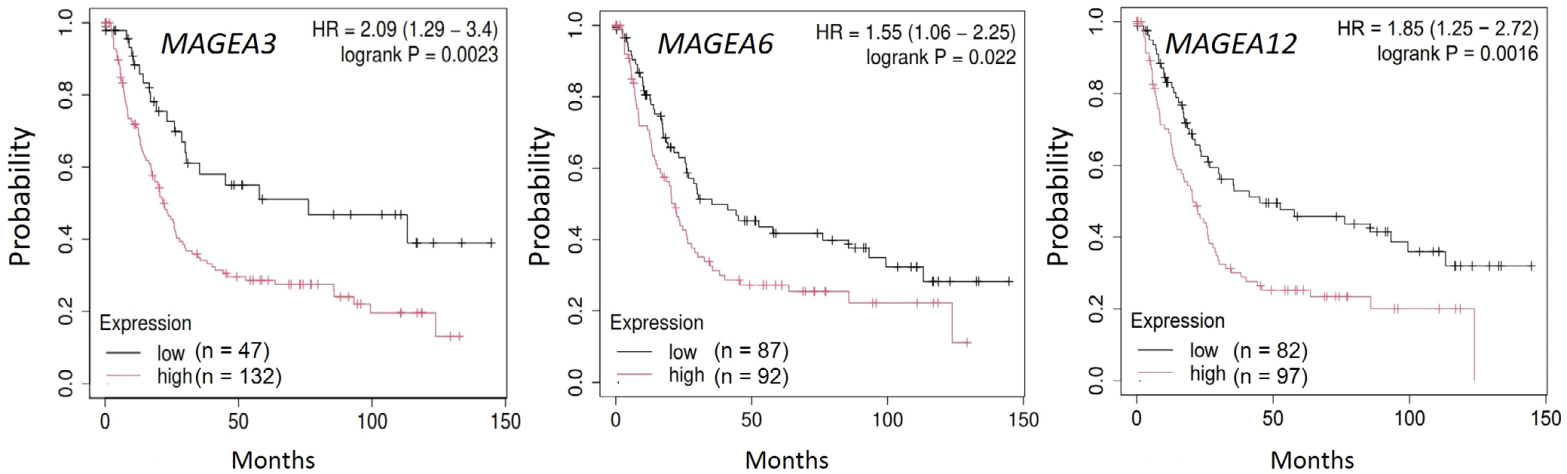
Intestinal‐type GC patients with lower *MAGEA3* (*p* = 0.0023) and *MAGEA12* (*p* = 0.0016) but not *MAGEA6* (*p* = 0.022) expression have improved overall survival in validation cohorts. Univariate analysis was performed using the km‐plotter.

The km plotter log‐rank test analysis (cohorts GSE 14210, 15,459, 22,377, 29,272 and 51,105) demonstrated similar patterns to the TCGA cohort, with intestinal‐type GC patients with lower *MAGEA3* (HR 2.09, 95% CI 1.29–3.4, *p* = 0.0023) and *MAGEA12* (HR 1.85, 95% CI 1.25–2.72, *p* = 0.0016) expression having better overall survival (Figure 5a,c). Expression of *MAGEA6* was not associated with survival outcomes (Figure 5b, HR 1.55, 95% CI 1.06–2.25, *p* = 0.022).

### 3.5 MAGEA Expression in Molecular GC Subtypes

To investigate the overlap between histological and molecular subtypes, the expression of MAGEA family genes was explored in the TCGA‐identified molecular subtypes of GC: Epstein–Barr virus (EBV), genomically stable (GS), microsatellite instability (MSI), and chromosomal instability (CIN).

**FIGURE 6:**
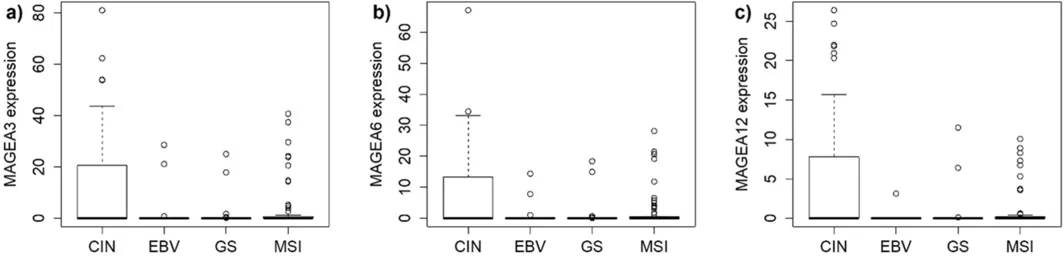
MAGEA family genes are highly expressed in the CIN molecular subtype. GC samples (*n* = 265) from the TCGA cohort were divided into molecular subtypes CIN, EBV, GS and MSI and assessed for differences in the expression of (a) *MAGEA3* (*p* = 10^−6^), (b) *MAGEA6* (*p* = 10^−7^), and (c) *MAGEA12* (*p* = 0.000073) using the Kruskal–Wallis rank sum test.

Expression by TCGA molecular subtype shows that higher expression of *MAGEA3* (*p* = 10^−6^), *MAGEA6* (*p* = 10^−7^), and *MAGEA12* (*p* = 0.000073) was seen mainly in the CIN subtype of GC, and to a lesser degree in the MSI subtype (Figure 6).

## 4 Discussion

MAGEA family members have been identified as promising targets for GC immunotherapy; however, trials have failed to demonstrate a benefit to patient survival. Since GC is heterogeneous and patient subpopulations rather than all GC patients may benefit from immunotherapeutics targeting MAGEAs, we determined whether the expression levels of MAGEA family members varied in different subtypes of GC.

MAGEA gene expression was analyzed in an Australian GC cohort with extensive follow‐up data, allowing correlation with long‐term survival. We found MAGEA family genes to be expressed in intestinal‐type GC and to a lesser extent in mixed GC. This finding was also observed in the TCGA cohort. High MAGEA gene expression was also found in GCs of the CIN molecular subtype. Our analysis identified that higher expression of MAGEA family genes in intestinal‐type GC patients was associated with poorer survival. On multivariate analysis of the MAUGIC cohort, high expression of *MAGEA3, MAGEA6*, and *MAGEA12* was associated with poorer progression‐free survival, and although survival data were limited in the TCGA cohort, the univariate progression‐free survival results for *MAGEA3* and *MAGEA12* are also consistent with our data. In contrast to the MAUGIC cohort data, high expression of *MAGEA6* was not associated with poorer progression‐free survival in the TCGA cohort. This discrepancy may reflect differences in cohort size and composition, treatment regimens across countries and healthcare settings, and gene‐expression platforms and dichotomisation thresholds. These differences may influence both survival outcomes and the detection of prognostic associations. Intestinal‐type GC datasets with long‐term follow‐up data on km plotter showed similar patterns and were consistent with the TCGA cohort, indicating that the prognostic impact of *MAGEA6* may be modest and cohort‐dependent, requiring validation in a larger cohort using harmonized methods.

There have been promising results with cancer regression in trials of patients with metastatic disease using vaccines and engineered cytotoxic T lymphocytes against MAGEA family members, alone and in combination with other therapies [27, 36]. However, significant toxicities have also been demonstrated, including neurotoxicity due to unrecognized MAGEA expression in the brain [27, 30, 31, 32]. In the adjuvant setting, Phase III trials of MAGEA vaccines involving more than 2000 patients with non‐small cell lung cancer and melanoma disappointingly have shown no improvement in disease‐free survival [54, 55, 56]. One explanation for this finding could be that MAGEA may not be critical for cancer growth and survival; therefore, targeting them will not impact tumor growth. Another reason could relate to tumor heterogeneity, potentially with only some subclones of a tumor population being affected by the vaccine. The identification of a subset of intestinal‐type GC with MAGEA gene expression that has poorer survival suggests that this may be a valid avenue of treatment to explore for some GC patients in the future.

The identification of a subset of intestinal‐type GC patients with high MAGEA expression, associated with poorer survival, potentially also identifies a prognostic marker for intestinal‐type GC. We found a bimodal MAGEA expression pattern in intestinal‐type GC, which is consistent with the proposed on/off mechanism of expression regulation by promoter hypomethylation or histone acetylation [23, 28]. Most patients had expression levels near background (5 or less), and a small subset of tumors with high expression levels were identified. This distribution supports the biological plausibility of a discrete high‐expression subgroup with potential clinical relevance. Together, these findings support the existence of a biologically distinct MAGEA‐high subgroup in intestinal‐type gastric cancer, consistent with observations in independent datasets including TCGA and km plotter. The progression‐free survival cohort included clinically heterogeneous cases, which should be considered when interpreting survival associations.

The finding that MAGEA genes were predominantly expressed in intestinal‐type GC is consistent with a previous study that investigated MAGEA family protein expression in 1097 GC cases using an antibody that detects MAGEA‐1, 2, 3, 4, 6, 10, and 12. In this study, expression of MAGEA was associated with intestinal‐type GC and poorer overall survival, although this lost significance when pTNM stage was included in multivariate modeling [22]. The lack of significance was likely due to the exclusion of Laurén classification as a variable in the multivariate analysis. Multiple studies have found expression of MAGEA to be associated with increased lymph node metastasis, staging, and immune cell infiltrate in GC [25, 57, 58, 59]. Interestingly, only a few studies found MAGEA expression to be associated with poorer overall survival [24, 57, 59, 60, 61], which might be due to assessing GC without classification into histological subtypes or lack of discrimination between the MAGEA family members.

In contrast to most other studies, we did not identify a significant association between MAGEA expression and lymph node metastasis. This might be due to the lack of taking non‐biological variables into account for the survival analysis. Non‐biological variables that influence survival could be the adequacy of surgery, including the number of lymph nodes resected, patient co‐morbidities, and receipt of adjuvant chemotherapy or radiotherapy. However, determining progression‐free survival as an endpoint, rather than overall survival, excludes patients from the analysis who have died from causes other than cancer. This makes progression‐free survival a focused measure to understand the impact of MAGEA expression on cancer‐related outcomes, without being confounded by deaths from unrelated causes, which could dilute the findings. There is evidence that the likelihood of relapse after adjuvant chemotherapy is higher in diffuse‐type GC than in intestinal‐type GC [5], suggesting that adjuvant therapy could influence relapse patterns differently for diffuse‐type GC and intestinal‐type GC. A larger cohort would be needed to account for these differences.

Upon classification of GCs into molecular subtypes, we found high *MAGEA* gene expression in CIN GCs. This molecular subtype of GC contains mostly intestinal‐type GC cases; however, molecular subtypes EBV and MSI are also mainly composed of intestinal‐type GCs. CIN GCs display extensive genomic instability, including frequent chromosomal rearrangements, amplification, mutations, and aberrant epigenetic modifications [2]. In somatic tissue, MAGEA promoters are usually methylated, associated with silencing of MAGEA expression [62]. The expression of MAGEA in cancer is driven by hypomethylation and leads to increased cancer growth and resistance to chemotherapy [59, 62, 63]. CIN GCs are characterized by hypomethylation, whereas EBV and MSI GCs have a high‐methylation epigenome [64, 65]. The difference in methylation patterns in the molecular subtypes may explain our finding that the expression of MAGEA members is mainly isolated to intestinal‐type GCs with the CIN subtype but not in intestinal‐type GCs with the EBV or MSI subtype.

Our findings provide a rationale for investigating MAGEA as targets for intestinal‐type GC immunotherapy. A subpopulation of intestinal‐type GC patients in the MAUGIC cohort had high *MAGEA3, 6*, and *12* expression, associated with poorer survival. This patient population may benefit from MAGEA‐targeted immunotherapy. These findings were also observed (*MAGEA3* and *MAGEA12*) in the TCGA dataset, supporting further investigation of MAGEA expression as potential biomarkers and therapeutic targets in CIN GC could benefit from MAGEA immunotherapy.

Side effects of targeting MAGEA will have to be carefully considered and monitored. Targeting intestinal‐type GC or CIN GC with MAGEA therapeutics could help maximize treatment outcomes while sparing patients who are unlikely to respond.

## 5 Limitations

The survival analysis should be interpreted in the context of the relatively small number of patients with intestinal‐type GC available for analysis (*n* = 49), including only 16 patients classified as having high MAGEA expression. No prospective sample size or power calculation was performed because this was an exploratory retrospective analysis, and the *MAGEA3/6* and *MAGEA12* cut‐offs were selected based on visual inspection of density plots. Dichotomisation of continuous expression variables may also introduce threshold‐dependent bias.

The external analyses also have important limitations. In the TCGA dataset, incomplete follow‐up information limited the analysis to univariate PFS, preventing adjustment for clinical covariates as performed in our cohort. Furthermore, the km plotter platform does not permit application of the custom MAGEA expression cut‐offs used in our study, and therefore different dichotomisation criteria were applied. Consequently, these analyses provide supportive evidence for the observed associations but should not be considered strict independent validation.

Our findings are based on a historical cohort (accrual through 2009), with PFS evaluated in a cohort that included patients presenting with metastases at the time of surgery and those treated with palliative resections. In addition, the majority of patients in that era did not receive neoadjuvant or perioperative chemotherapy, a treatment strategy that has since become standard for locally advanced gastric cancer [66]. These factors may influence the generalizability of our findings to contemporary cohorts treated under modern protocols. Nevertheless, the cohort provides a valuable source of treatment‐naïve tumor tissue, which is increasingly difficult to obtain with the wider use of neoadjuvant treatment.

The multivariate analysis was restricted by the availability and completeness of clinical covariates, and residual confounding by treatment and other clinical factors cannot therefore be excluded. Although key findings were supported by examination of independent datasets, including TCGA and km‐plotter, further prospective studies in uniformly staged, contemporary cohorts are required to confirm the clinical utility of MAGEA expression in gastric cancer. Furthermore, our analyses are based on MAGEA mRNA expression without corresponding protein‐level validation. Thus, MAGEA should be considered a potential prognostic biomarker candidate rather than an established clinical biomarker. Larger, independently powered cohorts with pre‐specified expression thresholds, comprehensive clinical annotation, and protein‐level assessment will be required to establish the prognostic relevance of MAGEA expression in intestinal‐type GC.

## Author Contributions

**Silke Neumann:** writing – original draft, writing – review and editing, formal analysis. **Trevor Leong:** conceptualization, data curation, writing – review and editing. **Rita A. Busuttil:** conceptualization, methodology, writing – review and editing, formal analysis, funding acquisition, supervision. **Alex Boussioutas:** conceptualization, writing – review and editing, funding acquisition, supervision. **Sharon T. Pattison:** conceptualization, investigation, funding acquisition, writing – original draft, writing – review and editing, formal analysis, project administration.

## Funding

Collection and curation of the study cohort has been funded by National Health and Medical Research Council project grants (2004–2006 ID288714; 2007–2009 ID454584); 2009–2011 Cancer Australia ID570996; 2012–2014 Peter Mac Foundation grant IDAPP1030980. S. Pattison was supported by the Odlin Research Fellowship, Royal Australasian College of Physicians and a National Health and Medical Research Council post‐graduate scholarship APP1039393.

## Ethics Statement

All procedures followed were in accordance with the ethical standards of the responsible committee on human experimentation (institutional and national) and with the Helsinki Declaration of 1964 and later versions.

## Consent

Written informed consent was obtained from study participants, and ethical approval was obtained from Institutional Review Boards from individual hospitals associated with the study (Peter MacCallum Cancer Centre 12/25).

## Conflicts of Interest

The authors declare no conflicts of interest.

## Data Availability

Some of the data that support the findings of this study (overall survival) are openly available in km‐plotter at https://kmplot.com/analysis/ reference number [GSE51105]. The remaining data are available from the corresponding author, S.T. P., upon reasonable request.

## References

[1] F. Bray , M. Laversanne , H. Sung , et al., “Global Cancer Statistics 2022: GLOBOCAN Estimates of Incidence and Mortality Worldwide for 36 Cancers in 185 Countries,” CA: A Cancer Journal for Clinicians 74, no. 3 (2024): 229–263, 10.3322/caac.21834. 38572751

[2] Cancer Genome Atlas Research Consortium , “Comprehensive Molecular Characterization of Gastric Adenocarcinoma,” Nature 513, no. 7517 (2014): 202–209, 10.1038/nature13480. PMC4170219 25079317

[3] M. Biggar , S. Srinivasa , B. Wickramarachchi , R. Babor , G. H. Poole , and A. G. Hill , “Gastric Cancer Location and Histological Subtype in Pacific People and Maori Defies International Trends,” New Zealand Medical Journal 124, no. 1331 (2011): 39–44. 21725411

[4] S. Pattison , G. B. Mann , G. Crosthwaite , et al., “Predictors of Outcome After Surgery for Gastric Cancer in a Western Cohort,” ANZ Journal of Surgery 86, no. 6 (2016): 469–474, 10.1111/ans.12915. 25388659

[5] S. Pattison , C. Mitchell , S. Lade , T. Leong , R. A. Busuttil , and A. Boussioutas , “Early Relapses After Adjuvant Chemotherapy Suggests Primary Chemoresistance in Diffuse Gastric Cancer,” PLoS One 12, no. 9 (2017): e0183891, 10.1371/journal.pone.0183891. PMC5602536 28922362

[6] K. Wang , E. Li , R. A. Busuttil , et al., “A Cohort Study and Meta‐Analysis of the Evidence for Consideration of Lauren Subtype When Prescribing Adjuvant or Palliative Chemotherapy for Gastric Cancer,” Therapeutic Advances in Medical Oncology 12 (2020): 1758835920930359, 10.1177/1758835920930359. PMC7378722 32754227

[7] I. B. Tan , T. Ivanova , K. H. Lim , et al., “Intrinsic Subtypes of Gastric Cancer, Based on Gene Expression Pattern, Predict Survival and Respond Differently to Chemotherapy,” Gastroenterology 141, no. 2 (2011): 476–485, 10.1053/j.gastro.2011.04.042. PMC3152688 21684283

[8] Z. Lei , I. B. Tan , K. Das , et al., “Identification of Molecular Subtypes of Gastric Cancer With Different Responses to PI3‐Kinase Inhibitors and 5‐Fluorouracil,” Gastroenterology 145, no. 3 (2013): 554–565, 10.1053/j.gastro.2013.05.010. 23684942

[9] F. Berlth , E. Bollschweiler , U. Drebber , A. H. Hoelscher , and S. Moenig , “Pathohistological Classification Systems in Gastric Cancer: Diagnostic Relevance and Prognostic Value,” World Journal of Gastroenterology 20, no. 19 (2014): 5679–5684, 10.3748/wjg.v20.i19.5679. PMC4024777 24914328

[10] R. Cristescu , J. Lee , M. Nebozhyn , et al., “Molecular Analysis of Gastric Cancer Identifies Subtypes Associated With Distinct Clinical Outcomes,” Nature Medicine 21, no. 5 (2015): 449–456, 10.1038/nm.3850. 25894828

[11] A. Boussioutas , H. Li , J. Liu , et al., “Distinctive Patterns of Gene Expression in Premalignant Gastric Mucosa and Gastric Cancer,” Cancer Research 63, no. 10 (2003): 2569–2577. 12750281

[12] X. Chen , S. Y. Leung , S. T. Yuen , et al., “Variation in Gene Expression Patterns in Human Gastric Cancers,” Molecular Biology of the Cell 14, no. 8 (2003): 3208–3215, 10.1091/mbc.e02-12-0833. PMC181561 12925757

[13] S. T. Tay , S. H. Leong , K. Yu , et al., “A Combined Comparative Genomic Hybridization and Expression Microarray Analysis of Gastric Cancer Reveals Novel Molecular Subtypes,” Cancer Research 63, no. 12 (2003): 3309–3316. 12810664

[14] C. H. Ooi , T. Ivanova , J. Wu , et al., “Oncogenic Pathway Combinations Predict Clinical Prognosis in Gastric Cancer,” PLoS Genetics 5, no. 10 (2009): e1000676, 10.1371/journal.pgen.1000676. PMC2748685 19798449

[15] M. A. Shah , R. Khanin , L. Tang , et al., “Molecular Classification of Gastric Cancer: A New Paradigm,” Clinical Cancer Research: An Official Journal of the American Association for Cancer Research 17, no. 9 (2011): 2693–2701, 10.1158/1078-0432.Ccr-10-2203. PMC3100216 21430069

[16] P. Lauren , “The Two Histological Main Types of Gastric Carcinoma: Diffuse and So‐Called Intestinal‐Type Carcinoma. An Attempt at a Histo‐Clinical Classification,” Acta Pathologica et Microbiologica Scandinavica 64 (1965): 31–49. 10.1111/apm.1965.64.1.31 14320675

[17] C. S. Fuchs , M. Ozguroglu , Y. J. Bang , et al., “Pembrolizumab Versus Paclitaxel for Previously Treated PD‐L1‐Positive Advanced Gastric or Gastroesophageal Junction Cancer: 2‐Year Update of the Randomized Phase 3 KEYNOTE‐061 Trial,” Gastric Cancer: Official Journal of the International Gastric Cancer Association and the Japanese Gastric Cancer Association 25, no. 1 (2022): 197–206, 10.1007/s10120-021-01227-z. PMC8732941 34468869

[18] Y. Y. Janjigian , A. Kawazoe , Y. Bai , et al., “Pembrolizumab Plus Trastuzumab and Chemotherapy for HER2‐Positive Gastric or Gastro‐Oesophageal Junction Adenocarcinoma: Interim Analyses From the Phase 3 KEYNOTE‐811 Randomised Placebo‐Controlled Trial,” Lancet 402, no. 10418 (2023): 2197–2208, 10.1016/S0140-6736(23)02033-0. 37871604

[19] Y. K. Kang , L. T. Chen , M. H. Ryu , et al., “Nivolumab Plus Chemotherapy Versus Placebo Plus Chemotherapy in Patients With HER2‐Negative, Untreated, Unresectable Advanced or Recurrent Gastric or Gastro‐Oesophageal Junction Cancer (ATTRACTION‐4): A Randomised, Multicentre, Double‐Blind, Placebo‐Controlled, Phase 3 Trial,” Lancet Oncology 23 (2022): 234–247, 10.1016/S1470-2045(21)00692-6. 35030335

[20] F. Pietrantonio , G. Randon , M. Di Bartolomeo , et al., “Predictive Role of Microsatellite Instability for PD‐1 Blockade in Patients With Advanced Gastric Cancer: A Meta‐Analysis of Randomized Clinical Trials,” ESMO Open 6, no. 1 (2021): 100036, 10.1016/j.esmoop.2020.100036. PMC7815473 33460964

[21] E. De Plaen , K. Arden , C. Traversari , et al., “Structure, Chromosomal Localization, and Expression of 12 Genes of the MAGE Family,” Immunogenetics 40, no. 5 (1994): 360–369, 10.1007/bf01246677. 7927540

[22] E. J. Jung , M. A. Kim , H. S. Lee , et al., “Expression of Family A Melanoma Antigen in Human Gastric Carcinoma,” Anticancer Research 25, no. 3b (2005): 2105–2111. 16158951

[23] C. Xie , V. V. Subhash , A. Datta , et al., “Melanoma Associated Antigen (MAGE)‐A3 Promotes Cell Proliferation and Chemotherapeutic Drug Resistance in Gastric Cancer,” Cellular Oncology (Dordrecht) 39, no. 2 (2016): 175–186, 10.1007/s13402-015-0261-5. PMC13001867 26868260

[24] M. Endo , M. Kanda , K. Sawaki , et al., “Tissue Expression of Melanoma‐Associated Antigen A6 and Clinical Characteristics of Gastric Cancer,” Anticancer Research 39, no. 11 (2019): 5903–5910, 10.21873/anticanres.13794. 31704814

[25] J. Jin , J. Tu , J. Ren , et al., “Comprehensive Analysis to Identify MAGEA3 Expression Correlated With Immune Infiltrates and Lymph Node Metastasis in Gastric Cancer,” Frontiers in Oncology 11 (2021): 784925–784925, 10.3389/fonc.2021.784925. PMC8712941 34970496

[26] H. Inoue , M. Mori , J. Li , et al., “Human Esophageal Carcinomas Frequently Express the Tumor‐Rejection Antigens of MAGE Genes,” International Journal of Cancer 63, no. 4 (1995): 523–526, 10.1002/ijc.2910630411. 7591261

[27] D. W. Meek and L. Marcar , “MAGE‐A Antigens as Targets in Tumour Therapy,” Cancer Letters 324, no. 2 (2012): 126–132, 10.1016/j.canlet.2012.05.011. 22634429

[28] T. Honda , G. Tamura , T. Waki , et al., “Demethylation of MAGE Promoters During Gastric Cancer Progression,” British Journal of Cancer 90, no. 4 (2004): 838–843, 10.1038/sj.bjc.6601600. PMC2410179 14970862

[29] J. Vansteenkiste , M. Zilelinski , A. Linder , J. Dahabre , E. Esteban , and W. Malinowski , “Final Results of a Multi‐Center, Double‐Blind, Randomized, Placebo‐Controlled Phase II Study to Assess the Efficacy of MAGE‐A3 Immunotherapeutic as Adjuvant Therapy in Stage IB/II Non‐Small Cell Lung Cancer (NSCLC),” Journal of Clinical Oncology 25, no. 18 (2007): 7554. https://ascopubs.org/doi/10.1200/jco.2007.25.18_suppl.7554.

[30] R. A. Morgan , N. Chinnasamy , D. Abate‐Daga , et al., “Cancer Regression and Neurological Toxicity Following Anti‐MAGE‐A3 TCR Gene Therapy,” Journal of Immunotherapy 36, no. 2 (2013): 133–151, 10.1097/CJI.0b013e3182829903. PMC3581823 23377668

[31] D. Atanackovic , N. K. Altorki , Y. Cao , et al., “Booster Vaccination of Cancer Patients With MAGE‐A3 Protein Reveals Long‐Term Immunological Memory or Tolerance Depending on Priming,” Proceedings of the National Academy of Sciences of the United States of America 105, no. 5 (2008): 1650–1655, 10.1073/pnas.0707140104. PMC2234199 18216244

[32] D. Atanackovic , N. K. Altorki , E. Stockert , et al., “Vaccine‐Induced CD4+ T Cell Responses to MAGE‐3 Protein in Lung Cancer Patients,” Journal of Immunology 172, no. 5 (2004): 3289–3296, 10.4049/jimmunol.172.5.3289. 14978137

[33] Y.‐C. Lu , L. L. Parker , T. Lu , et al., “Treatment of Patients With Metastatic Cancer Using a Major Histocompatibility Complex Class II–Restricted T‐Cell Receptor Targeting the Cancer Germline Antigen MAGE‐A3,” Journal of Clinical Oncology: Official Journal of the American Society of Clinical Oncology 35, no. 29 (2017): 3322–3329, 10.1200/jco.2017.74.5463. PMC5652397 28809608

[34] D. S. Hong , B. A. V. Tine , A. J. Olszanski , et al., “Phase I Dose Escalation and Expansion Trial to Assess the Safety and Efficacy of ADP‐A2M4 SPEAR T Cells in Advanced Solid Tumors,” Journal of Clinical Oncology: Official Journal of the American Society of Clinical Oncology 38, no. 15 (2020): 102, 10.1200/JCO.2020.38.15_suppl.102.

[35] D. K. Krishnadas , S. Shusterman , F. Bai , et al., “A Phase I Trial Combining Decitabine/Dendritic Cell Vaccine Targeting MAGE‐A1, MAGE‐A3 and NY‐ESO‐1 for Children With Relapsed or Therapy‐Refractory Neuroblastoma and Sarcoma,” Cancer Immunology, Immunotherapy: CII 64, no. 10 (2015): 1251–1260, 10.1007/s00262-015-1731-3. PMC11028635 26105625

[36] A. Alsalloum , J. A. Shevchenko , and S. Sennikov , “The Melanoma‐Associated Antigen Family A (MAGE‐A): A Promising Target for Cancer Immunotherapy?,” Cancers (Basel) 15, no. 6 (2023): 1779, 10.3390/cancers15061779. PMC10046478 36980665

[37] S. P. D'Angelo , D. M. Araujo , A. R. Abdul Razak , et al., “Afamitresgene Autoleucel for Advanced Synovial Sarcoma and Myxoid Round Cell Liposarcoma (SPEARHEAD‐1): An International, Open‐Label, Phase 2 Trial,” Lancet 403, no. 10435 (2024): 1460–1471, 10.1016/S0140-6736(24)00319-2. PMC11419333 38554725

[38] F. L. Greene , D. L. Page , I. D. Fleming , et al., eds., AJCC Cancer Staging Manual, 6th ed. (Springer, 2002).

[39] T. Barrett , S. E. Wilhite , P. Ledoux , et al., “NCBI GEO: Archive for Functional Genomics Data Sets–Update,” Nucleic Acids Research 41, no. Database issue (2013): 991–995, 10.1093/nar/gks1193. PMC3531084 23193258

[40] R. A. Busuttil , J. George , R. W. Tothill , et al., “A Signature Predicting Poor Prognosis in Gastric and Ovarian Cancer Represents a Coordinated Macrophage and Stromal Response,” Clinical Cancer Research: An Official Journal of the American Association for Cancer Research 20, no. 10 (2014): 2761–2772, 10.1158/1078-0432.Ccr-13-3049. 24658156

[41] V. Chalifa‐Caspi , I. Yanai , R. Ophir , et al., “GeneAnnot: Comprehensive Two‐Way Linking Between Oligonucleotide Array Probesets and GeneCards Genes,” Bioinformatics (Oxford, England) 20, no. 9 (2004): 1457–1458, 10.1093/bioinformatics/bth081. 14962929

[42] E. Cerami , J. Gao , U. Dogrusoz , et al., “The cBio Cancer Genomics Portal: An Open Platform for Exploring Multidimensional Cancer Genomics Data,” Cancer Discovery 2, no. 5 (2012): 401–404, 10.1158/2159-8290.CD-12-0095. PMC3956037 22588877

[43] J. Gao , B. A. Aksoy , U. Dogrusoz , et al., “Integrative Analysis of Complex Cancer Genomics and Clinical Profiles Using the cBioPortal,” Science Signaling 6, no. 269 (2013): pl1, 10.1126/scisignal.2004088. PMC4160307 23550210

[44] L. Gautier , L. Cope , B. M. Bolstad , and R. A. Irizarry , “Affy–Analysis of Affymetrix GeneChip Data at the Probe Level,” Bioinformatics (Oxford, England) 20, no. 3 (2004): 307–315, 10.1093/bioinformatics/btg405. 14960456

[45] R. C. Gentleman , V. J. Carey , D. M. Bates , et al., “Bioconductor: Open Software Development for Computational Biology and Bioinformatics,” Genome Biology 5, no. 10 (2004): R80, 10.1186/gb-2004-5-10-r80. PMC545600 15461798

[46] G. K. Smyth , “Linear Models and Empirical Bayes Methods for Assessing Differential Expression in Microarray Experiments,” Statistical Applications in Genetics and Molecular Biology 3 (2004): 3, 10.2202/1544-6115.1027. 16646809

[47] G. K. Smyth , “Limma: Linear Models for Microarray Data,” in Bioinformatics and Computational Biology Solutions Using R and Bioconductor, ed. R. Gentleman , V. J. Carey , W. Huber , R. A. Irizarry , and S. Dudoit (Springer New York, 2005), 397–420.

[48] R. A. Irizarry , B. Hobbs , F. Collin , et al., “Exploration, Normalization, and Summaries of High Density Oligonucleotide Array Probe Level Data,” Biostatistics 4, no. 2 (2003): 249–264, 10.1093/biostatistics/4.2.249. 12925520

[49] Y. Benjamini and Y. Hochberg , “Controlling the False Discovery Rate: A Practical and Powerful Approach to Multiple Testing,” Journal of the Royal Statistical Society, Series B: Statistical Methodology 57, no. 1 (1995): 289–300.

[50] T. M. Therneau , Modeling Survival Data: Extending the Cox Model (Springer, 2000).

[51] “*A Package for Survival Analysis in S,”* (2015), http://CRAN.R‐project.org/package=survival.

[52] B. Győrffy , “Integrated Analysis of Public Datasets for the Discovery and Validation of Survival‐Associated Genes in Solid Tumors,” Innovation (Camb) 5, no. 3 (2024): 100625, 10.1016/j.xinn.2024.100625. PMC11066458 38706955

[53] A. M. Szász , A. Lánczky , Á. Nagy , et al., “Cross‐Validation of Survival Associated Biomarkers in Gastric Cancer Using Transcriptomic Data of 1,065 Patients,” Oncotarget 7, no. 31 (2016): 49322–49333, 10.18632/oncotarget.10337. PMC5226511 27384994

[54] B. Dreno , J. F. Thompson , B. M. Smithers , et al., “MAGE‐A3 Immunotherapeutic as Adjuvant Therapy for Patients With Resected, MAGE‐A3‐Positive, Stage III Melanoma (DERMA): A Double‐Blind, Randomised, Placebo‐Controlled, Phase 3 Trial,” Lancet Oncology 19, no. 7 (2018): 916–929, 10.1016/S1470-2045(18)30254-7. 29908991

[55] J. F. Vansteenkiste , B. C. Cho , T. Vanakesa , et al., “Efficacy of the MAGE‐A3 Cancer Immunotherapeutic as Adjuvant Therapy in Patients With Resected MAGE‐A3‐Positive Non‐Small‐Cell Lung Cancer (MAGRIT): A Randomised, Double‐Blind, Placebo‐Controlled, Phase 3 Trial,” Lancet Oncology 17, no. 6 (2016): 822–835, 10.1016/s1470-2045(16)00099-1. 27132212

[56] P. Therasse , J. F. Vansteenkiste , M. Zielinski , et al., “MAGRIT Phase III Trial: MAGE‐A3 Antigen‐Specific Cancer Immunotherapy (ASCI) as Adjuvant Therapy in Patients With Completely Resected Stage IB‐IIIA NSCLC,” Journal of Clinical Oncology: Official Journal of the American Society of Clinical Oncology 29, no. 15 (2011): TPS210, 10.1200/jco.2011.29.15_suppl.tps210.

[57] C. H. Jeon , I. H. Kim , and H. D. Chae , “Prognostic Value of Genetic Detection Using CEA and MAGE in Peritoneal Washes With Gastric Carcinoma After Curative Resection: Result of a 3‐Year Follow‐Up,” Medicine 93, no. 11 (2014): e83, 10.1097/md.0000000000000083. PMC4616273 25192488

[58] K. Ogata , R. Aihara , E. Mochiki , et al., “Clinical Significance of Melanoma Antigen‐Encoding Gene‐1 (MAGE‐1) Expression and Its Correlation With Poor Prognosis in Differentiated Advanced Gastric Cancer,” Annals of Surgical Oncology 18, no. 4 (2011): 1195–1203, 10.1245/s10434-010-1399-z. 21042944

[59] Y. Lian , M. Sang , L. Gu , et al., “MAGE‐A Family Is Involved in Gastric Cancer Progression and Indicates Poor Prognosis of Gastric Cancer Patients,” Pathology, Research and Practice 213 (2017): 943–948, 10.1016/j.prp.2017.05.007. 28647208

[60] Z. W. Wang , Q. Y. Yu , M. J. Xu , C. Y. Zhou , J. P. Li , and X. H. Liao , “MAGE‐A11 Is a Potential Prognostic Biomarker and Immunotherapeutic Target in Gastric Cancer,” Aging (Albany NY) 16, no. 1 (2024): 285–298, 10.18632/aging.205368. PMC10817374 38180746

[61] Q. Jia , X. Xian , Y. Li , J. Mu , and Z. Du , “Research Progress in Effects of MAGE‐A Family on Gastric Cancer,” Zhong Nan Da Xue Xue Bao. Yi Xue Ban 48, no. 2 (2023): 260–267, 10.11817/j.issn.1672-7347.2023.220042. PMC10930336 36999473

[62] Y. Lian , L. Meng , P. Ding , and M. Sang , “Epigenetic Regulation of MAGE Family in Human Cancer Progression‐DNA Methylation, Histone Modification, and Non‐Coding RNAs,” Clinical Epigenetics 10, no. 1 (2018): 115, 10.1186/s13148-018-0550-8. PMC6126015 30185218

[63] A. Colemon , T. M. Harris , and S. Ramanathan , “DNA Hypomethylation Drives Changes in MAGE‐A Gene Expression Resulting in Alteration of Proliferative Status of Cells,” Genes and Environment 42 (2020): 24, 10.1186/s41021-020-00162-2. PMC7392716 32760472

[64] R. Xiang and T. Fu , “Gastrointestinal Adenocarcinoma Analysis Identifies Promoter Methylation‐Based Cancer Subtypes and Signatures,” Scientific Reports 10, no. 1 (2020): 21234, 10.1038/s41598-020-78228-y. PMC7719188 33277583

[65] G. Usui , K. Matsusaka , Y. Mano , et al., “DNA Methylation and Genetic Aberrations in Gastric Cancer,” Digestion 102, no. 1 (2021): 25–32, 10.1159/000511243. 33070127

[66] F. Lordick , F. Carneiro , S. Cascinu , et al., “Gastric Cancer: ESMO Clinical Practice Guideline for Diagnosis, Treatment and Follow‐Up,” Annals of Oncology 33, no. 10 (2022): 1005–1020, 10.1016/j.annonc.2022.07.004. 35914639

